# Enhancement of Self-Management of Metabolic Syndrome Among Adults in Urban, Low-Income Settings of India Using Digital Health Interventions: Protocol for a Mixed Methods Study

**DOI:** 10.2196/40144

**Published:** 2025-06-23

**Authors:** Ashish Joshi, Ashoo Grover, Usha Agrawal, Harpreet Kaur, Bhavya Malhotra, Sandeep Agrawal

**Affiliations:** 1 School of Public Health University of Memphis Memphis, TN United States; 2 Indian Council of Medical Research New Delhi India; 3 National Institute of Pathology New Delhi India; 4 Foundation of Healthcare Technologies Society New Delhi India

**Keywords:** digital health intervention, metabolic syndrome, cardiometabolic risk factors, mixed methods, self-management, urban lifestyle, low-income setting, poverty, community health, India, health promotion

## Abstract

**Background:**

Metabolic syndrome (MetS) is a growing concern among adult populations in India, particularly among those living in urban, low-income settings. This group is challenged by a combination of risk factors, including an urbanized lifestyle, poor access to health care, and financial limitations, leading to high levels of obesity, diabetes, and hypertension.

**Objective:**

This study aims to address this challenge by designing, developing, and piloting a tailored, mobile-enabled, interactive, digital health intervention to enhance self-management of MetS among individuals living in urban, low-income settings in New Delhi, India.

**Methods:**

The study uses mixed methods, including both quantitative and qualitative data collection, to design and evaluate the effectiveness of the intervention built on a multifactorial model in improving the self-management of MetS. Data will be collected at baseline and 12 months from adults living in urban, low-income settings in New Delhi. The results will contribute to our understanding of the interplay of risk factors in MetS and the impact of tailored digital health interventions in addressing this challenge. The findings will be disseminated to both national and international audiences through peer-reviewed publications.

**Results:**

This study was funded in March 2022 for 3 years. The project started in April 2022. Data collection began in June 2022. The results are expected to be published in 2025.

**Conclusions:**

The study is expected to provide valuable insights into the role of digital health interventions in enhancing the self-management of MetS among urban, low-income populations.

**International Registered Report Identifier (IRRID):**

PRR1-10.2196/40144

## Introduction

### Background and Rationale

Metabolic syndrome (MetS), also known as “Syndrome X,” poses a growing threat to public health. By 2020, cardiovascular diseases (CVDs) are projected to be the leading cause of death and disability in India, with 2.6 million deaths expected [1]. Meanwhile, stroke, a major contributor to CVD, is projected to increase to 894 per 100,000 people [2]. MetS is characterized by a cluster of metabolic factors including high blood pressure, impaired glucose tolerance or diabetes, and dyslipidemia (elevated levels of triglycerides and low concentration of high-density lipoproteins) marked by the presence of abdominal obesity [3]. The specific etiology of MetS remains unclear, but the interplay between genetic, behavioral, clinical, and environmental factors such as urbanization, mechanization, and rural-to-urban migration play an important role in its etiology [1,3-5].

Although there are numerous studies on the effects of health interventions for nutrition and physical activity on a single risk factor such as obesity, CVD, and diabetes [6,7], there have been no studies evaluating the impact of multifactorial interventions. The role of social determinants of health in reducing the burden of MetS has not been considered in these studies. As preventive measures, changes through intervention in dietary habits and physical activity are key lifestyle changes recommended by medical literature [8-10]. Despite increased public awareness about noncommunicable diseases (NCDs), the burden of NCDs has not decreased.

The individual behaviors of residents of urban, low-income settings in their environment are crucial in understanding the causes of NCDs and cannot be tackled through policy recommendations based on lumped quantitative data alone. Such methods ignore the interplay of culture, politics, poverty, economics, and actions in urban, low-income conditions and the role of the Sustainable Development Goals [11]. Given the concentration of health programs and policy resources in cities, it is assumed that city inhabitants have more access to services like rehabilitation and health care, and that people with low income living in cities are thus better off than their rural counterparts. This is where the vulnerability of individuals residing in urban, low-income settings arises; the issue of equality becomes critical for urban individuals with low income, who face complicated and debilitating issues such as the inability to pay for products and services, and a lack of social support structures. Urban individuals with low income also have economic hurdles when it comes to making healthier dietary decisions. The accessibility, availability, and affordability of ultraprocessed, high-sugar, high-fat foods in contrast to fresh fruits, vegetables, and meat lead to a high risk for MetS. A few barriers to telehealth interventions identified in the Indian context stand true for urban, low-income populations, such as the digital divide, language barriers, perceived usefulness or preference for face-to-face consultation, perceived ease of use, etc [12]. Food environments, food security, sanitation and hygiene, clean drinking water, etc, are contributing factors to MetS among those residing in urban, low-income settings [13,14].

Prior studies found that individuals treated for diabetes or hypertension only often received medications and do not adopt lifestyle changes such as consuming a healthier diet and increasing physical activity [15]. There is a dearth of effective intervention models that are feasible in Indian, rural, low-resource settings, which face significant challenges owing to poverty, low literacy, and gender norms [16]. Interventions to address social, behavioral, and environmental determinants of MetS and cardiometabolic disorders are less developed [17]. Hence, the creation of science-based, behavior-focused, context-specific, and culturally relevant interventions that take into account the social determinants of health is the solution to address the burden of MetS. Therefore, the proposed study aims to fill existing gaps in understanding how cardiometabolic and environmental factors interact and influence the self-management of MetS among urban, low-income populations.

### Study Aim and Objectives

This study aims to design, develop, and pilot-test an interactive, tailored, internet- and mobile-enabled health intervention to enhance the self-management of MetS among individuals living in urban, low-income settings.

The study aims to fulfill the following 3 objectives:

1. To develop a model that examines cardiometabolic risk factors such as diet, physical activity, stress, and sleep and their interplay with the living environment in facilitating the self-management of MetS among adults living in urban, low-income settings in New Delhi;
2. To design and develop an interactive digital health intervention to enhance the self-management of MetS based on the findings of objective 1; and
3. To pilot-test the utility and feasibility of the proposed digital health intervention among adults living in urban, low-income settings to help them self-manage their MetS.

## Methods

### Study Design and Population

To achieve objective 1, a mixed method approach comprising qualitative and quantitative assessments will be used to develop a model that will describe the interplay between the living environment and other cardiometabolic risk factors such as diet, physical activity, stress, and sleep. In-depth interviews will be conducted among a sample population of 120 participants enrolled from urban, low-income settings across 4 zones of New Delhi (North, South, East, and West).

To achieve objective 2, a quasi-experimental study design will be used with a sample of 80 participants, using a human-centered approach to design and develop an interactive, tailored, mobile- and internet-enabled digital health intervention. The findings of objective 1 will help define the essential elements and features of the proposed digital health intervention.

**Figure 1:**
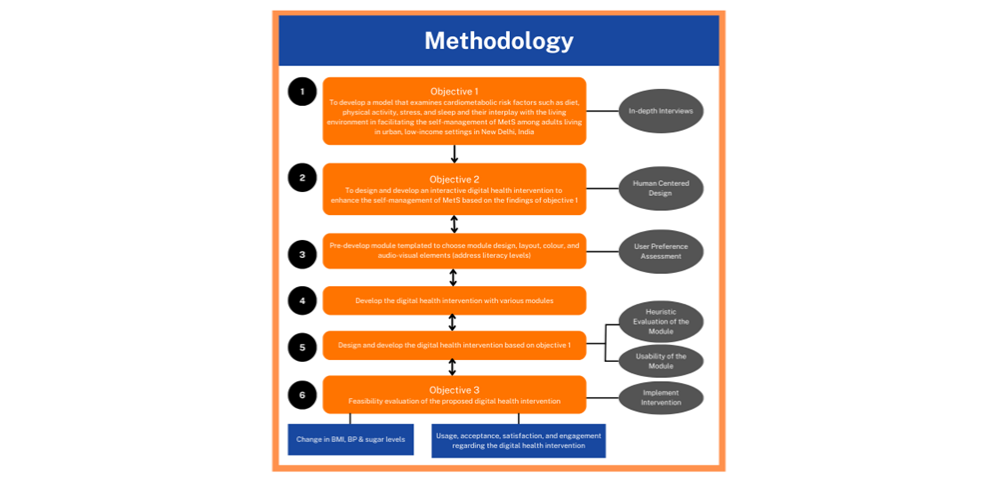
Flowchart of study methodology. BP: blood pressure; MetS: metabolic syndrome.

Objective 3 of the study will be to pilot-test the utility and feasibility of the proposed digital health intervention and to measure usage, adherence, satisfaction, and acceptance regarding the proposed digital health intervention. For this, a sample size of 30 people will be enrolled from each of the 8 urban, low-income areas (240 participants) of New Delhi. The methodology is presented graphically in Figure 1.

### Inclusion and Exclusion Criteria

Eligible participants will include adults (aged 18 years and older) who provided informed consent, have an Android phone, are willing to use technology, are available for follow-up interviews, and have been diagnosed with MetS (presence of 3 of the 5 risk factors of MetS). Individuals who are aged <18 years; have a terminal illness, impaired cognition, presence of coexisting illness documented in the medical record (type 1 diabetes), or self-reported systemic inflammation (eg, rheumatoid arthritis or acute systemic infection); are pregnant or lactating; are involved in other clinical trials or protocols related to MetS; or fail to adhere to study procedures (eg, scheduled surgery, travel plans, or scheduling difficulties that do not permit full participation) will be excluded from the study.

### Assessment Variables

The full text of the survey instrument is presented in Multimedia Appendix 1.

#### Sociodemographic Profile

Data regarding age, income level, employment status, education level, smoking, and alcohol use will be collected. Information about prior use of computers, their frequency of usage, prior use of the internet, and sources of health information will also be captured.

#### Medical History

The medical history of the participant will be gathered by taking in medical records of the participant, asking for the history of identified NCDs, and taking note of prescriptions and medications used.

#### Laboratory Assessment

Blood sugar, hemoglobin A_1c_, and blood pressure levels will be assessed at baseline and after 12 months.

#### Anthropometric Status

Height, weight, and waist circumference will be collected using a standard technique. BMI will be computed from height and weight measurements.

#### 24-Hour Dietary Recall

A standard protocol will be used to assess the dietary intake of the previous 24 hours. The methodology will include visual aids (photographs of servings) to assist them in accurately reporting food intake.

#### Dietary Diversity Questionnaire

The Food and Agriculture Organization’s Individual Dietary Diversity Score tool [18] will be used to assess diversity in the diets of the individual participants; the tool comprises 16 food groups, of which 9 will be analyzed. Diets comprising foods from ≤3 groups will be categorized as having a low diversity, diets with 4-5 food groups will be categorized as moderate diversity, and those >6 groups will be categorized as diets of high diversity.

#### Perceived Stress Scale

The Perceived Stress Scale [19] is the most widely used psychological instrument for measuring the perception of stress. It is a measure of the degree to which situations are appraised as stressful.

#### Physical Activity Assessment

Participants’ physical activity will be assessed using the Global Physical Activity Questionnaire [20]. The questionnaire will calculate the total time spent in physical activity for recreation, occupation, household work, and transportation in the last 7 days.

#### MEDFICTS Dietary Assessment Questionnaire

The MEDFICTS Dietary Assessment Questionnaire [21] consists of 8 food categories: meat, eggs, dairy, fried foods, fat in baked goods, convenience foods, fats added at the table, and snacks. It is an efficient tool to quickly assess the adherence of participants to the fat components of a diet and identify those consuming a diet higher in total fat, saturated fat, and cholesterol.

#### Satisfaction With the Digital Health Intervention

Satisfaction with the digital health intervention will be measured using the Client Satisfaction Questionnaire–8 Question [22]. This 8-item questionnaire is a self-report statement of satisfaction with the intervention.

#### Acceptance of the Digital Health Intervention

The Technology Acceptance Questionnaire will be used to measure acceptance of the digital health intervention. It comprises 25 questions based on the Technology Acceptance Model with six constructs: (1) perceived ease of use, (2) perceived usefulness, (3) attitude, (4) behavioral intention, (5) self-efficacy, and (6) subjective norm.

#### Engagement With the Digital Health Intervention

Engagement with the digital health intervention will be measured by the number of weekly log-ins, prompt completion of tasks, and submission of their day-to-day data.

#### The Pittsburgh Sleep Quality Index

The Pittsburgh Sleep Quality Index [23] is a self-rated questionnaire that will be used to assess sleep quality and disturbances over a 1-month time interval. Together, 19 individual items generate 7 “component” scores: subjective sleep quality, sleep latency, sleep duration, habitual sleep efficiency, sleep disturbances, use of sleeping medication, and daytime dysfunction. The sum of the scores for these 7 components yields a single global score.

#### System Usability Scale

The System Usability Scale [24] assesses the appropriateness of the functionality and usability of the digital health intervention. It also assesses the extent to which users view the application as supporting their goals and tasks, as well as checks the interface’s usability.

#### Health Knowledge, Attitude, and Practice Assessment

The Health Knowledge, Attitude, and Practice Assessment measures the knowledge of MetS, risk factors of comorbidities, attitude to health-related choices, and practice patterns. It is an informative tool, and the measured items are bound to change with time. Deploying the assessment at different time points can help identify key changes and effects of the intervention.

#### Adherence to Medication

The Medication Adherence Report Scale [25] will be used as a self-reported tool to assess patient adherence to prescribed medication through 5 key questions about forgetfulness, dosage changes, interruption, skipping, and underuse.

#### Summary of Diabetes Self-Care Activities

Information regarding self-care activities among patients with diabetes will be collected using the revised version of the Summary Diabetes and Hypertension Self-Care Activities Questionnaire [26]. The core questionnaire components, along with a few more questions concerning each of the components and items on medication practices, will be assessed.

### Data Analysis Plan

The gathered variable outcome data will be checked for inconsistencies or missing values and will be confidentially stored and retrieved for analysis. The qualitative (in-depth interviews) data will be analyzed by content analyses. Descriptive analyses will report the means and SDs of the data results. 2-tailed *t* tests will be used to compare the means between categorical and continuous variables. Chi-square analysis will be used to analyze categorical variables. Univariate and bivariate analyses of independent variables will be done. Reporting of results will be done at 95% CI, and *P*<.05 used.

### Project Timelines

The detailed project timeline and schedule corresponding to the study objectives are presented in Table 1.

**Table 1:** Study timeline.

Objectives, study phases, and tasks				Month							
			1		2-3	4-6	7-14	15-20	31-32	32-33	34-36
**Preparatory phase**											
	**1**										
		Stakeholder meeting	✓								
		In-depth interviews			✓	✓					
		Analysis of qualitative, in-depth interview data				✓					
**Intervention development**											
	**2**										
		Design and development of human-centered intervention					✓				
		Heuristic evaluation					✓				
		Usability evaluation of the proposed system					✓				
		Refine the proposed, digital health, self-management intervention					✓				
	**3**										
		Final deployment of the proposed, digital health, self-management intervention						✓			
**Data collection**											
	**4**										
		Study recruitment						✓			
		Baseline and follow-up data collection						✓	✓		
	**5**										
		Statistical analysis						✓	✓	✓	
		Report writing and manuscript preparation								✓	✓

### Quality Assurance

Data collection and entry will be done by trained field staff following standard techniques and protocols. Training sessions for the field workers will be held to acquaint the data collection team with the study aim, objectives, study area, and inclusion/exclusion criteria. Information will also be given for study instruments, anthropometric data collection, in-depth interviews (engagement with the respondent and probing), and informed consent (for data collection, audio recording, and pictures). To ensure efficiency and high-quality data collection and processing, weekly meetings with the field and research staff will be held. A log of all data collected will be maintained, and weekly data checks will be done.

### Ethical Considerations

The study was approved by the Indian Council of Medical Research—National Institute of Pathology Institutional Human Ethics Committee in January 2022 (approval NIP-IEC/29-12-2021/05/01R1). The study will be conducted according to the Declaration of Helsinki, as it involves human participants [19]. Written informed consent to participate in the study will be obtained from each of the selected participants. Audio recording of consent will be done for individuals with limited education. Complete data confidentiality and anonymity of study participants will be ensured. Study consent will be available in English and a local Indian dialect. Data will be collected by trained data collectors electronically using tablet computers and saved on the servers.

### Expected Outcomes

The study outcomes include the enhancement of self-management of MetS among individuals living in urban and low-income slum settings. The study will identify influencing risk factors on MetS and examine the role of digital interventions to address the risks. The authors also expect the study to increase self-efficacy and the knowledge, attitudes, and practices related to self-management of diabetes, hypertension, and other risk factors of MetS among individuals living in urban, low-income settings. The study results will be disseminated at various levels, including scientific journal publications, conference proceedings, etc.

## Results

This study was funded in March 2022 for 3 years. The project started in April 2022. Data collection began in June 2022. The results are expected to be published in 2025.

## Discussion

The paper aims to address the growing threat of MetS in India, where despite numerous studies on the effects of health interventions, there have been no evaluations of multifactorial interventions, and the role of social determinants of health in reducing the burden of MetS has not been considered. The proposed digital health intervention model will use the principles of human-centered approach, involving users’ input throughout the design and development of the intervention. The authors aim to fill the gap in understanding the interaction between cardiometabolic and environmental factors and self-management of MetS among urban, low-income populations. The study results are expected to increase the knowledge, attitudes, and practices related to self-management of MetS and other risk factors among urban, low-income populations. However, the study has limitations, such as being limited to urban, low-income populations only and facing challenges in implementing interventions, which may limit its generalizability. The findings from the study will contribute to understanding of the role of digital health interventions in enhancing the self-management of MetS, which will greatly impact public health at large.

## Appendix

Multimedia Appendix 1 Full text of the survey questionnaire.

## Abbreviations

CVD: cardiovascular disease
MetS: metabolic syndrome
NCD: noncommunicable disease

## Data Availability

The data supporting this study’s findings are available upon request from the corresponding author.
