# Supplementary material for: Enhancement of Self-Management of Metabolic Syndrome Among Adults in Urban, Low-Income Settings of India Using Digital Health Interventions: Protocol for a Mixed Methods Study
Source: JMIR Res Protoc. 2025 Jun 23;14:e40144. doi: 10.2196/40144 (PMC12235204; doi:10.2196/40144)
Supplement: Multimedia Appendix 1 [file resprot_v14i1e40144_app1.docx]

## Multimedia Appendix 1

# Full text of the survey questionnaire.

# SURVEY CATEGORIES:

| **Annexure Number** | **Annexure Title** |
| --- | --- |
|  | Informed consent form |
| 1. | Socio-demographic profile, Medical history and Anthropometric status |
| 2. | 24 hour Dietary Recall |
| 3. | Dietary Diversity Questionnaire |
| 4. | Perceived Stress Scale |
| 5. | Global physical activity questionnaire |
| 6. | MEDFICTS Dietary Assessment Questionnaire |
| 7. | CSQ-Client Satisfaction Questionnaire |
| 8. | The Pittsburgh Sleep Quality Index |
| 9. | System Usability Scale (SUS) |
| 10. | Health Knowledge, Attitude and Practice Assessment |
| 11. | Medication adherence Questionnaire |
| 12. | The Summary of Diabetes Self-Care Activities |

# Written Consent form:

**Note:** Audio recording of consent will be done for uneducated individuals

**Title:** Digital Health Intervention to enhance self-management of Metabolic Syndrome (MetS) among adults living in urban poor settings

**PI**: Dr. Ashoo Grover

**Objective**: The objective of the proposed study is to enhance self-management of MetS among urban poor by digital intervention using mobile/internet enabled platform.

**Informed Consent**

I understand that I am being asked to participate in this study to facilitate the researchers to develop a model that to enhance self-management of Metabolic Syndrome (MetS) among adults living in urban poor settings

I understand that the study will involve my participation and follow-up for a period of 12 months. I understand that my participation in the study is voluntary and that it requires me to provide information on my socio demographics, dietary habits, physical activity, and sleep and stress level on regular basis using a mobile platform. I understand that I will have follow up assessments at months 1, 3, 6, and 9 and 12 using a series of questionnaires related to above mentioned parameters. Clinical examination will be done for the parameter BP and blood sugar. Anthropometric assessment will be to measure height, weight and waist circumference. .

The information provided will be used for study purposes and the results of the study will be included in journal publication and the funding agencies and may be presented in the conferences but will not include any identifiers. The participation in the study is voluntary.

Signature of Field Staff: _____________________

Name of the Respondent with signature: ____________________

Thumb Impression __________________

Audio recording consent needed: Yes/No

If yes Audio recording of consent taken: Yes/No

Mobile/Phone: ____________________ Address:_________________________________________________________

______________________________________________________________________

Date: ________________________

Signature

Thumb impression

**Enrolment No**

**Study Title:** Digital Health Intervention to enhance self-management of Metabolic Syndrome (MetS) among adults living in urban poor settings

# Annexure 1: Socio-demographic profile, Medical history and Anthropometric status

**A1. Code _____________________**

**A2. Date** __ __ (d) __ __ (m) __ __ __ __ (y)

**A3.** Name of the respondent _________________________________________

**A4.** Location of Slum--------------------------------

| **S. No.** | **Question** | **Response** |
| --- | --- | --- |
| A5. | Gender | 1. Male 2. Female |
| A6. | Age of the respondent | Years and month or only years |
| A7. | Religion |  |
| A8. | Marital status | 1. Married 2. Unmarried 3. Separated 4. Divorced 5. Others |
| A9. | Educational qualification | 1. Uneducated 2. Primary school (I-V standard) 3. Middle school (VI-X standard) 4. High school (XI-XII standard) 5. Intermediate or diploma 6. Graduate 7. Profession or Honours |
| A10. | Occupation | 1. Unemployed 2. Elementary occupation 3. Plant and machine operators and assemblers 4. Craft and related trade workers 5. Skilled agricultural and fishery workers 6. Skilled workers and shops and market sales workers 7. Clerks 8. Technicians and associated professionals 9. Professionals 10. Legislators, senior officials and managers |
| A11. | Monthly household income (Rupees) | 1. < 3,907 2. 3,908-11,707 3. 11,708-19,515 4. 19,516-29,199 5. 29,200-39,032 6. 39,033-78,062 7. >78,063 |

**B. Anthropometric measurements of the respondent**

|  | **Measurement** | **Reading 1** | | **Reading 2** |
| --- | --- | --- | --- | --- |
| B.1 | Weight Kg |  | |  |
| B.2 | Height cm |  | |  |
| B.3 | Waist Circumference |  | |  |
| B.4 | BMI |  |  | |

**C. Familiarity with technology**

| C.1 | Do you use mobile phone? | 1. Yes 2. No |
| --- | --- | --- |
| C.2 | Do you have Internet access? | 1. Yes 2. No |
| C.3 | Do you know how to read a text message? | 1. Yes 2. No |
| C.4 | Do you know how to send a text message? | 1. Yes 2. No |

**D. Clinical Assessment**

|  | Measurement | Reading |
| --- | --- | --- |
| D.1 | Fasting/Random Blood Glucose |  |
| D.2 | HbA1C |  |
| D.3 | Blood Pressure |  |

**E. Clinical history**

| E.1 | Earlier Diagnosed with diabetes | 1. Yes 2. No 3. Don’t know |
| --- | --- | --- |
|  | If yes |  |
|  | E.1a Duration of diabetes |  |
|  | E.1b Recent blood sugar report |  |
| E.2 | Type of medication | 1. Medicine 2. Insulin 3. Any other |
| E.3 | Earlier Diagnosed with hypertension | 1. Yes 2. No 3. Don’t know |
| E.4 | Diagnosed with any other NCD (cancer, overweight, obesity, cardio vascular diseases) | 1. Yes 2. No 3. Don’t know |
| E.5 | Do you smoke? | 1. Yes 2. No |
| E.6 | Do you drink? | 1. Yes 2. No |
| E. 7 | Do you exercise? | 1. Yes 2. No 3. Sometimes |
|  | E.7b How many days per week? |  |
|  | E.7c Duration of exercise |  |
|  | E.7d Type of work |  |
|  | E.7e Travel time for work per day |  |
|  | E.7f Time spent on other recreational activities |  |
| E. 8 | Do you use any type of drugs? | 1. Yes 2. No 3. Sometimes |

F. Health Behavior

F.1 Tobacco Consumption

F.1a Have you ever used tobacco or used smokeless tobacco (chewing tobacco, snuff, khaini)?

1. In the past 2. Present 3. Never

If in the present move to question 5.4,

If Never, Move to Alcohol Consumption.

F.1b How long ago did you stop using (smoke, sniff or chew) tobacco products?

1. ____ Years ago 2._____ Months ago 3._____Can’t Say

F.1c What tobacco product were you using? What was your average daily consumption? (bidis, cigarettes, hukkah, cigars, cheroots, smokeless tobacco, khaini, snuff etc.)

Tobacco Product Daily Consumption (units consumed per day)

1.__________ ____

2.__________ ____

3. __________ ____

F.1d For how long have you been smoking or using tobacco daily?

1. ____ Years ago 2._____ Months ago 3._____Can’t Say

F.1 e What tobacco product are you using? What is your average daily consumption?

(bidis, cigarettes, hukkah, cigars, cheroots, smokeless tobacco, khaini, snuff etc.)

Tobacco Product Daily Consumption (units consumed per day)

1.__________ ____

2.__________ ____

3. __________ ____

F.2 . Alcohol Consumption

F.2a Have you ever consumed a drink that contains alcohol (such as beer, wine, spirits, etc.)?

1. In the past 2. Present 3. Never

If ‘in the present’ move to next annexure

If ‘never’ move to next question

F.2b How long ago did you stop consuming alcohol beverages?

1. ____ Years ago 2._____ Months ago 3._____Can’t Say

F.2c What alcohol beverages were you using? What was your average daily consumption?

(wine, bear, rum, whisky etc.)

Alcohol Product Daily Consumption

__________ ____

__________ ____

__________ ____

F.2d For how long have you been consuming alcohol beverages?

1. ____ Years ago 2._____ Months ago 3._____Can’t Say

F.2e What alcohol beverages are you using? What is your average daily consumption?

(wine, bear, rum, whisky etc.)

Alcohol Product Daily Consumption

__________ ____

__________ ____

__________ ____

# Annexure 2: 24 hour Dietary Recall

G.1 a) DATE

b) DAY:

INSTRUCTIONS: Please recall what you have eaten in the last 24 hours giving information about the entire main and the in-between meals.

| Meal and timings | Menu | Amount in household measures  (g) | Ingredients | Amount in household measures  (g) | Amount  (g) | Comments(Size, Thickness,  Greasiness, Consistency, Brand) |
| --- | --- | --- | --- | --- | --- | --- |
| Early Morning |  |  |  |  |  |  |
| Breakfast |  |  |  |  |  |  |
| Mid-Morning |  |  |  |  |  |  |
| Lunch |  |  |  |  |  |  |
| Evening tea |  |  |  |  |  |  |
| Dinner |  |  |  |  |  |  |
| Post dinner |  |  |  |  |  |  |

# Annexure 3: DIETARY DIVERSITY QUESTIONNAIRE Capital or small letter

Please describe the foods (meals and snacks) that you ate yesterday during the day and night, whether at home or outside the home. Start with the first food eaten in the morning. Write down all food and drinks mentioned by the respondent. When the respondent has finished, probe for meals and snacks not mentioned.

H.1

| Breakfast | Snack | Lunch | Snack | Dinner | Snack |
| --- | --- | --- | --- | --- | --- |
|  |  |  |  |  |  |

H.2

| Question | Food Categories | Examples | No = 0 Yes = 1 |
| --- | --- | --- | --- |
| 1. | CEREALS | corn/maize, rice, wheat, sorghum, millet or any other grains or foods made from these ( chapatti, rice , bajra cheela, halwa, porridge) |  |
| 2. | WHITE ROOTS AND TUBERS | Potatoes, Turnip, raddish |  |
| 3. | VITAMIN A RICH VEGETABLES AND TUBERS | pumpkin, carrot, squash, or sweet potato that are orange inside + other locally available vitamin A rich vegetables (e.g. red sweet pepper) |  |
| 4. | DARK GREEN LEAFY VEGETABLES | dark green leafy vegetables, including wild forms + locally available vitamin A rich leaves such as amaranth, kale, spinach, fenugreek leaves, mustard leaves, |  |
| 5. | OTHER VEGETABLES | other vegetables (e.g. tomato, onion, eggplant, Cauliflower, cucumber, onion, peas, brinjal, bitter gourd , bottle gourd,) + other locally available vegetables |  |
| 6. | VITAMIN A RICH FRUITS | ripe mango, apricot (fresh or dried), ripe papaya, dried peach, and 100% fruit juice made from these + other locally available vitamin A rich fruits |  |
| 7. | OTHER FRUITS | Apple, banana , gooseberry, guava,lemon, sapota, orange , lime |  |
| 8. | ORGAN MEAT | liver, kidney, heart or other organ meats or blood-based foods |  |
| 9. | FLESH MEATS | beef, pork, lamb, goat, rabbit, game, chicken, duck, other birds, insects |  |
| 10. | EGGS | eggs from chicken, duck, guinea fowl or any other egg |  |
| 11. | FISH AND SEAFOOD | fresh or dried fish or shellfish |  |
| 12. | LEGUMES, NUTS AND SEEDS | Chickpea, cowpea, broad beans, horsegram, lentils, moong dal, peas, soyabean, peanuts, almonds, walnuts, sesame, flaxseeds |  |
| 13. | MILK AND MILK PRODUCTS | milk, buttermilk , paneer, curd , khoa |  |
| 14. | OILS AND FATS | oil, fats or butter added to food or used for cooking |  |
| 15. | RED PALM PRODUCTS | oil, fats or butter added to food or used for cooking |  |
| 16. | SWEETS | sugar, honey, sweetened soda or sweetened juice drinks, sugary foods such as chocolates, candies, cookies and cakes |  |
| 17. | SPICES, CONDIMENTS, BEVERAGES | spices (black pepper, salt), condiments (soy sauce, hot sauce), coffee, tea, alcoholic beverages |  |
| 18. | SAVOURY AND FRIED SNACKS | Samosa, tikki, kachori, pakora, bhatura, purri |  |
|  |  |  |  |
|  | Did you eat anything (meal or snack) OUTSIDE the home yesterday? |  |  |

# Annexure 4 : PERCEIVED STRESS SCALE

A more precise measure of personal stress can be determined by using a variety of instruments that have been designed to help measure individual stress levels. The first of these is called the Perceived Stress Scale.

The Perceived Stress Scale (PSS) is a classic stress assessment instrument. The tool, while originally developed in 1983, remains a popular choice for helping us understand how different situations affect our feelings and our perceived stress. The questions in this scale ask about your feelings and thoughts during the last month. In each case, you will be asked to indicate how often you felt or thought a certain way. Although some of the questions are similar, there are differences between them and you should treat each one as a separate question. The best approach is to answer fairly quickly. That is, don’t try to count up the number of times you felt a particular way; rather indicate the alternative that seems like a reasonable estimate.

For each question choose from the following alternatives:0 – never, 1 - almost never, 2 - sometimes , 3 - fairly often , 4 - veryoften

I.1. In the last month, how often have you been upset because of something that happened unexpectedly?_______

I.2. In the last month, how often have you felt that you were unable to control the important things in your life?_______

I.3. In the last month, how often have you felt nervous and stressed? _______

I4. In the last month, how often have you felt confident about your ability to handle your personal problems? _______

I.5. In the last month, how often have you felt that things were going your way? _______

I.6. In the last month, how often have you found that you could not cope with all the things that you had to do? _______

I.7. In the last month, how often have you been able to control irritations in your life? _______

I.8. In the last month, how often have you felt that you were on top of things? _______

I.9. In the last month, how often have you been angered because of things that happened that were outside of your control? ______

I.10. In the last month, how often have you felt difficulties were piling up so high that you could not overcome them? _______

# Annexure 5: GLOBAL PHYSICAL ACTIVITY QUESTIONNAIRE

|  | | | |
| --- | --- | --- | --- |
| **Questions** | | **Response** | **Code** |
| **J.A. Activity at work** | | | |
| 1. | Does your work involve vigorous- intensity activity that causes large increase in breathing or heart rate like [Carrying or lifting heavy loads, digging or construction work] for at least 10 minutes continuously? | **Yes 1**  **No 2**  **(If No, go to P 4)** | **P1** |
| 2. | In a typical week, on how many days do you do vigorous- intensity activities as part of your work? | **Number of days** | **P2** |
| 3. | How much time do you spend doing vigorous- intensity activities at work on a typical day? | **Hours Minutes** | **P3**  **(a-b)** |
| 4. | Does your work involve moderate- intensity activity that causes small increases in breathing or heart rate such as brisk walking [or carrying light loads] for at least 10 minutes continuously? | **Yes**  **No if no, go to P 7)** | **P4** |

| 5. | In a typical week, or how many days do you do moderate- intensity activities as part of your work? | **Number of Days** | **P5** |
| --- | --- | --- | --- |
| 6. | How much time do you spend doing moderate- intensity activities at work on a typical day? | **Hours Minutes** | **P6**  **(a-b)** |
| **J.B. Travel to and from places** | | | |
| 7. | Do you walk or use a bicycle (pedal cycle) for at least 10 minutes continuously to get to and from places? | **Yes 1**  **No 2**  **(If No, go to P 10)** | **P7** |
| 8. | In a typical week, on how many days do you walk or bicycle for at least 10 minutes continuously to get to and from places? | **Number of Days** | **P8** |
| 9. | How much time do you spend walking or bicycling for travel on a typical day? | **Hours Minutes** | **P9** |
| **J. C. Recreational Activities** | | | |
| The next questions exclude the work and transport activities that you have already mentioned.  Now I would like to ask you about sports, fitness and recreational activities (leisure), [insert relevant terms]. | | | |
| 10. | Do you do any vigorous-intensity sports, fitness or recreational (leisure) activities that cause large increase in breathing or heart rate like [running or football,] for at least 10 minutes continuously? | **Yes**  **No**  **(if No, go to p 13)** | **P10** |

| 11. | In a typical week, on how many days do you do vigorous- intensity sports, fitness or recreational (leisure) activities? | **Number of days** | **P11** |
| --- | --- | --- | --- |
| 12. | How much time do you spend doing vigorous- intensity sports, fitness or recreational activities on a typical day? | **Hours Minutes** | **P12**  **(a-b)** |
| **Questions** | | **Response** | **Code** |
| 13. | In a typical week, on how many days do you do  moderate- intensity sports, fitness or recreational (leisure) activities? | **Yes**  **No**  **(if No, go to P 16)** | **P13** |
| 14. | In a typical week, on how many days do you do moderate-intensity sports, fitness or recreational (leisure) activities? | **Number of days** | **P14** |
| 15. | How much time do you spend doing moderate- intensity sports, fitness or recreational (leisure) activities on a typical day? | **Hours Minutes** | **P15** |
| **J. D. Sedentary Behavior** | | | |
| The following question is about sitting or reclining at work, at home, getting to and from places, or with friends including time spent [sitting at a desk, sitting with friends, travelling in car, bus, train, reading, playing cards or watching television], but do not include time spent sleeping. | | | |
| 16. | How much time do you usually spend sitting or reclining on a typical day? | **Hours Minutes** | **P16**  **(a-b)** |

# Annexure 6: MEDFICTS Dietary Assessment Questionnaire

To fill out the MEDFICTS nutrition questionnaire, you will make two check marks for each row. Check one of the blank boxes under the “weekly consumption” category indicating how often per week you eat those foods listed to the left. Then check one box under the “Serving Size” category indicating the typical serving size, or amount of that food you eat at a time. That’s all there is to it! Don’t worry about the scoring or anything else on the form, just hand it back to the people at the front desk.

| **Food Category** | **Weekly Consumption** | | | |  | **Serving Size** | | | | **Score** |
| --- | --- | --- | --- | --- | --- | --- | --- | --- | --- | --- |
|  | Rarely/ Never | | 3 or less | 4 or more |  | Small  <5 oz/d  1 pt | Average  5 oz/d  2 pts | | Large  >5 oz/d  3 pts |  |
| **K.1 Meats:**   - Recommended amount per day: ≤5 oz (equal in size to 2 decks of playing cards) - Base your estimate on the food you consume most often - Beef and lamb selections are trimmed to 1/8” fat | | | | | | | | | | |
| **Group 1:** 10gm or more total fat in 3oz cooked portion   1. **Beef –** Ground beef, Ribs, Steak (T-bone, Flank, Porterhouse, Tenderloin), Chuck blade roast, Brisket, Meatloaf (W/ ground beef), Corned beef 2. **Processed meats –** ¼ lb burger or lrg. Sandwich, Bacon, Lunch meat, Sausage/Knockwurst, Hot dogs, Ham (bone-end), Ground turkey 3. **Other meats, Poultry, Seafood –** pork chops (center loin), pork roast    1. (Blade, Boston, Sirloin), Pork spareribs, ground pork, lamb chops, lamb (ribs), organ meats, chicken w/skin, eel, mackerel, pompano | |  |  |  | X |  |  | |  |  |
|  |  | 0 pts | 3 pts | 7 pts |  | 1 pt | 2 pts | | 3 pts |  |
| **Group 2:** Less than 10 gm total fat in 3oz cooked portion   1. **Lean Beef –** Round steak (eye of round, Top round), sirloin, Tip & bottom round, chuck arm pot roast, Top Loin 2. **Low fat processed meats –** Low fat lunch meat, Canadian bacon, “lean” fast food sandwich, boneless ham 3. **Other meats, poultry, seafood –** chicken, turkey (w/out skin), most seafood, lamb leg shank, pork tenderloin, sirloin top loin, veal cutlets, sirloin, shoulder, ground veal, venison, veal chops and ribs, lamb (whole leg, loin, fore-shank, sirloin) | |  |  |  | X |  |  | |  |  |
|  |  | n/a | n/a | n/a |  | n/a | n/a | | 6 pts total |  |
|  | | | | | | | | | | |
| **K. 2 Eggs –** Weekly consumption is the number of times you eat eggs each week Check the number of eggs eaten each time | | | | | | | | | | |
| **Group 1:** whole eggs, yolks | |  |  |  | X | ≤1 egg | 2 eggs | ≥3 eggs | |  |
|  |  |  |  |  |  |  |  |  | |  |
|  |  | 0 pts | 3 pts | 7 pts |  | 1 pt | 2 pts | 3 pts | |  |
| **Group 2:** Egg whites, Egg substitutes (1/2 cup) | |  |  |  | X |  |  |  | |  |
|  | | | | | | | | | | |
| **K.3 Dairy** | | | | | | | | | | |
| **Milk –** Average serving 1 cup  **Group 1:** Whole milk, 2% milk, 2% buttermilk, Yogurt (whole milk) | |  |  |  | X |  |  | |  |  |
|  |  | 0 pts | 3 pts | 7 pts |  | 1 pt | 2 pts | | 3 pts |  |
| **Group 2:** Fat-free milk, 1% milk, Fat-free buttermilk, Yogurt (Fat-free, 1% low fat) | |  |  |  | X |  |  | |  |  |
| **Cheese –** Average serving 1 oz  **Group 1:** Cream cheese, cheddar, Monterey Jack, Colby, Swiss, American processed, blue cheese, Regular cottage cheese (1/2 Cup), and Ricotta (1/4 cup) | |  |  |  | X |  |  | |  |  |
|  |  | 0 pts | 3 pts | 7 pts |  | 1 pt | 2 pts | | 3 pts |  |
| **Group 2:** Low-fat and fat-free cheeses, fat-free milk mozzarella, string cheese, low-  fat, fat-free milk, and fat-free cottage cheese (1/2 cup) and ricotta (1/4 cup) | |  |  |  | X |  |  | |  |  |

| **K.4 Frozen Desserts –** Average serving ½ cup | | | | | | | | |
| --- | --- | --- | --- | --- | --- | --- | --- | --- |
| **Group 1:** ice cream, milk shakes |  |  |  | X |  |  |  |  |
|  | 0 pts | 3 pts | 7 pts |  | 1 pt | 2 pts | 3 pts |  |
| **Group 2:** Low-fat ice cream, frozen yogurt |  |  |  | X |  |  |  |  |
|  | | | | | | | | |
| **K.5 Frying foods –** Average servings: see below. This section refers to method of preparation for vegetables and meat. | | | | | | | | |
| **Group 1:** French fries, fried vegetables (1/2 c), fried chicken, fish, meat (3 oz) |  |  |  | X |  |  |  |  |
|  | 0 pts | 3 pts | 7 pts |  | 1 pt | 2 pts | 3 pts |  |
| **Group 2:** Vegetables, not deep fried (1/2 cup), Meat, Poultry, or fish – prepared by baking, broiling, grilling, poaching, roasting, stewing: (3 oz) |  |  |  | X |  |  |  |  |
|  | | | | | | | | |
| **K.6 In Baked Goods** – 1 Average Serving | | | | | | | | |
| **Group 1**: Doughnuts, biscuits, butter rolls, muffins, croissants, sweet rolls, Danish, cakes, pies, coffee cakes, cookies |  |  |  | X |  |  |  |  |
|  | 0 pts | 3 pts | 7 pts |  | 1 pt | 2 pts | 3 pts |  |
| **Group 2**: fruit bars, low-fat cookies/cakes/pastries, angel food cake, home-made  baked goods with vegetable oils, breads, bagels |  |  |  | X |  |  |  |  |
|  | | | | | | | | |
| **K.7 Convenience Foods** | | | | | | | | |
| **Group 1**: Canned, Packaged, or Frozen dinners: e.g., Pizza (1slice), Macaroni &  Cheese (1 cup), Pot pie (1), cream soups (1 cup), Potato, rice & pasta dishes with cream/cheese sauces (1/2 cup) |  |  |  | X |  |  |  |  |
|  | 0 pts | 3 pts | 7 pts |  | 1 pt | 2 pts | 3 pts |  |
| **Group 2**: Diet/reduced calorie or reduced fat dinners (1), Potato, rice and pasta  dishes without cream/cheese sauces (1/2 cup). |  |  |  | X |  |  |  |  |
|  | | | | | | | | |
| **K.8 Table Fats** – Average serving 1 tbsp | | | | | | | | |
| **Group 1**: butter, stick margarine, regular salad dressing, mayonnaise, sour cream (2 Tbsp) |  |  |  | X |  |  |  |  |
|  | 0 pts | 3 pts | 7 pts |  | 1 pt | 2 pts | 3 pts |  |
| **Group 2**: Diet and tub margarine, low-fat and fat-free salad dressing, low-fat and fat  free mayonnaise |  |  |  | X |  |  |  |  |
|  | | | | | | | | |
| **K.9 Snacks** | | | | | | | | |
| **Group 1**: Chips (potato, corn, taco), cheese puffs, snack mix, nuts (1oz), regular  crackers (1/2 oz), candy (milk chocolate, caramel, coconut) about 1 ½ oz), regular popcorn (3 cups). |  |  |  | X |  |  |  |  |
|  | 0 pts | 3 pts | 7 pts |  | 1 pt | 2 pts | 3 pts |  |
| **Group 2**: Pretzels, fat-free chips (1oz), low fat crackers (1/2 oz), fruit rolls, licorice, hard candy (1 med piece), bread sticks (1-2 pcs), Air-popped or low-fat pop corn (3  cups). |  |  |  | X |  |  |  |  |

***MEDFICTS** = **M**eats, **E**ggs, **D**airy, **F**rying Foods, **I**n baked goods, **C**onvenience foods, **T**able fats, **S**nack

To Score: For each food category, multiply points in weekly consumption box by points in serving size box and record total in score column. If Group 2 foods checked, no points are scored (except Group 2 meats, large serving = 6 pts).

| **KEY:** |  | |
| --- | --- | --- |
|  | ≥70 | Need to make some dietary changes |
|  | 40 – 70 | Heart-Healthy diet |
|  | <40 | TLC Diet |

# Annexure 7 CSQ-8, CLIENT SATISFACTION QUESTIONNAIRE

Please help us improve our program by answering some questions about the services you have received. We are interested in your honest opinions, whether they are positive or negative.

*Please answer all of the questions.* We also welcome your comments and suggestions.

Thank you very much, we really appreciate your help.

**CIRCLE YOUR ANSWERS**

K.1 How would you rate the quality of service you have received?

4 3 2 1

*Excellent Good Fair Poor*

K.2 Did you get the kind of service you wanted?

1 2 3 4

*No, definitely not No, not really Yes, generally Yes, definitely*

K.3 To what extent has our program met your needs?

4 3 2 1

*Almost all of my need Most of my needs Only a few of need None of my needs*

*have been met have been met have been met have been met*

K.4 If a friend were in need of similar help, would you recommend our program to him or her?

1 2 3 4

*No, definitely not No, I don’t think so Yes, I think so Yes, definitely*

K.5 How satisfied are you with the amount of help you have received?

1 2 3 4

*Quite dissatisfied Indifferent or mildly dissatisfied Mostly satisfied Very satisfied*

K.6 Have the services you received helped you to deal more effectively with your problems?

4 3 2 1 *Yes, they helped a Yes, they helped No, they really No, they seemed togreat deal somewhat* *didn’t help make things worse*

K.7 In an overall, general sense, how satisfied are you with the service you have received?

4 3 2 1

*Very satisfied Mostly satisfied Indifferent or mildly Quite*

*Dissatisfied dissatisfied*

K.8 If you were to seek help again, would you come back to our program?

1 2 3 4

*No, definitely not No, I don’t think so Yes, I think so Yes, definitely*

# Annexure 8 - The Pittsburgh Sleep Quality Index

**Instructions:**

The following questions relate to your usual sleep habits during the past month *only*. Your answers should indicate the most accurate reply for the *majority* of days and nights in the past month. Please answer all the questions.

L.1 During the past month, when have you usually gone to bed at night?

**usual bed time**

L.2 During the past month, how long (in minutes) has it usually taken you to fall asleep each night?

**number of minutes**

L.3 During the past month, when have you usually got up in the morning?

**usual getting up time**

L.4 During the past month, how many hours of *actual* sleep did you get at night? (This may be different than the number of hours you spend in bed).

**hours of sleep per night**

For each of the remaining questions, check the one best response. Please answer *all* questions.

L.5 During the past month, how often have you had trouble sleeping because you……

1. Cannot get to sleep within 30 minutes

Not during the Less than Once or three or more

past month once a week twice a week times a week

1. Wake up in the middle of the night or early morning

Not during the Less than Once or Three or more

past month once a week twice a week times a week

1. Have to get up to use the bathroom

Not during the Less than Once or three or more

past month once a week twice a week times a week

1. Cannot breathe comfortably

Not during the Less than Once or three or more

past month once a week twice a week times a week

1. Cough or snore loudly

Not during the Less than Once or three or more

past month once a week twice a week times a week

1. Feel too cold

Not during the Less than Once or three or more

past month once a week twice a week times a week

1. Feel too hot

Not during the Less than Once or three or more

past month once a week twice a week times a week

1. Had bad dreams

Not during the Less than Once or three or more

past month once a week twice a week times a week

1. Have pain

Not during the Less than Once or three or more

past month once a week twice a week times a week

1. Other reason(s), please describe

How often during the past month have you had trouble sleeping because of this?

Not during the Less than Once or three or more

past month once a week twice a week times a week

L.8 During the past month, how would you rate your sleep quality overall?

Very good

Fairly good

Fairly bad

Very bad

1. During the past month, how often have you taken medicine (prescribed or “ over

the counter”) to help you sleep?

Not during the Less than Once or three or more

past month once a week twice a week times a week

1. During the past month, how often have you had trouble staying awake while

driving, eating meals, or engaging in social activity?

Not during the Less than Once or three or more

past month once a week twice a week times a week

1. During the past month, how much of a problem has it been for you to keep up

enough enthusiasm to get things done?

No problem at all

Only a very slight problem

Somewhat of a problem

A very big problem

1. Do you have a bed partner or roommate?

No bed partner or roommate

Partner/roommate in other room

Partner in same room, but not same bed

Partner in same bed

1. How often do you feel tired during the following times during the day?

*Morning*:

0 1 2 3

most days often occasionally never

*Afternoon*:

0 1 2 3

most days often occasionally never

*Evening*:

0 1 2 3

most days often occasionally never

# Annexure 9: System Usability Scale (SUS)

This is a standard questionnaire that measures the overall usability of a system. Please select the answer that best expresses how you feel about each statement after using the website today.

|  | **Strongly Disagree** | **Somewhat Disagree** | **Neutral** | **Somewhat Agree** | **Strongly Agree** |
| --- | --- | --- | --- | --- | --- |
| 1. **I think I would like to use this tool frequently.** |  |  |  |  |  |
| 1. **I found the tool unnecessarily complex.** |  |  |  |  |  |
| 1. **I thought the tool was easy to use.** |  |  |  |  |  |
| 1. **I think that I would need the support of a technical person to be able to use this system.** |  |  |  |  |  |
| 1. **I found the various functions in this tool were well integrated.** |  |  |  |  |  |
| 1. **I thought there was too much inconsistency in this tool.** |  |  |  |  |  |
| 1. **I would imagine that most people would learn to use this tool very quickly.** |  |  |  |  |  |
| 1. **I found the tool very cumbersome to use.** |  |  |  |  |  |
| 1. **I felt very confident using the tool.** |  |  |  |  |  |
| 1. **I needed to learn a lot of things before I could get going with this tool.** |  |  |  |  |  |

How likely are you to recommend this website to others? (please circle your answer)

Not at all likely 0 1 2 3 4 5 6 7 8 9 10 Extremely likely

# Annexure 10: Health Knowledge, Attitude and Practice Assessment

| **O.1. What is Metabolic Syndrome?? (select all that apply)**  High Blood Sugar Obesity High Cholesterol  Hypertension All of the above None of the above |
| --- |
| **O.2What are the risk factors of Diabetes and Hypertension? (select all that apply)**  Increasing Age Alcohol consumption Sedentary lifestyle  Increased carbohydrate All of the above None of the above  and Fat intake |
| **O.3** **Is family history a risk factor of diabetes and hypertension?**  Yes No Not Sure |
| **O.4 Is smoking a risk factor of heart disease?**  Yes No Not Sure |
| **O.5 How often do you consume fruits?**  Once a day Once a week Once a month Rarely Never |
| **O. 6 Do you think consumption of butter and ghee should be kept to a minimum?**  Yes No Not Sure |
| **O.7 What oil do you currently use to cook your food? (select all that apply)**  Ghee Vanaspati Sunflower oil Groundnut oil  Other I Don’t know |
| **O.8 Do you think we should restrict goods high in sodium?**  Yes No I Don’t know |
| **O.9 What is the recommended time gap between two frequent meals?**  Every 1 hour Every 3-4 hours Every 8 hours I don’t know |
| **O. 10 Do you keep a record of the diet you take?**  Yes No No one told me to do that I don’t know how to do it |
| **O.11 How frequently do you exercise?**  Everyday Often Sometimes Rarely Never |
| **O.12 What is a good amount of average physical activity on a daily basis?**  30-60 minutes of brisk walking About 2 hours of brisk walking I don’t know |
| **O.13 What are the most common reasons for you to be not physically active? (Select all that applies)**  I don’t have time to work out Exercise is boring I am too self-conscious to work out  I am too tired after work I get too discouraged All of the above  None of the above |
| **O.14 Have you heard the term Body Mass Index (BMI)?**  Yes No Not Sure |
| **O.15 Who is an obese person in the following, identify from the Body Mass Index**  **given?**  ≥25-29.9 kg/m^2^ ≥30 kg/m^2^ I don’t know |
| **O.16 Please select the right formula for calculating Body Mass Index.**  Suppose your weight is 70 kg and height is 5 feet 6 inch, then your BMI will be:  Weight = 70 kg, Height = 5’6” = 66 inch = 164.56 cm = 1.65 m  Your BMI will be  BMI = 70 (kg)  1.65 (m2)  BMI = 1.65(m2)  70 (kg)  I don’t know |
| **O.17 What value of Waist to Hip Ratio is a high risk for males?**  0.81-0.85 >0.85 > 1.00 I don’t know |
| **O. 18 Which individual is pre hypertensive?**  Systolic Blood Pressure ≥120 mmHg Diastolic Blood Pressure ≥80 mmHg  Systolic Blood Pressure ≥120 mmHg and Diastolic Blood Pressure ≥80 mmHg  All of the above I don’t know |
| **O. 19 What is considered to be abnormal fasting blood sugar value?**  ≥110 mg/dl ≥140 mg/dl None of the above I don’t know |
| **O.20 Please select the normal cholesterol values?**  <200 mg/dl <120 mg/dl <250 mg/dl None of the above I don’t know |

**Annexure 11: Medication adherence Questionnaire**

In the Medication adherence report scale **(**MARS-5), the following five questions will be asked to the respondents:

1. Do you forget to take medication?

2. Do you change the dosage of medication?

3. Do you stop taking medication for a while?

4. Do you skip taking medication?

5. Do you use medication less than prescribed?

# Annexure 12: The Summary of Diabetes Self-Care Activities

The questions below ask you about your diabetes self-care activities during the past 7 days. If you were sick during the past 7 days, please think back to the last 7 days that you were not sick.

**Q.1 Diet**

- 1. How many of the last SEVEN DAYS have you followed a healthful eating plan?

0 1 2 3 4 5 6 7

- 1. On average, over the past month, how many DAYS PER WEEK have you followed your eating plan?

0 1 2 3 4 5 6 7

- 1. On how many of the last SEVEN DAYS did you eat five or more servings of fruits and vegetables?

0 1 2 3 4 5 6 7

- 1. On how many of the last SEVEN DAYS did you eat high fat foods such as red meat or full-fat dairy products?

0 1 2 3 4 5 6 7

**Q.2 Exercise**

- - - - 1. On how many of the last SEVEN DAYS did you participate in at least 30 minutes of physical activity? (Total minutes of contin-uous activity, including walking).

0 1 2 3 4 5 6 7

- - - - 1. On how many of the last SEVEN DAYS did you participate in a specific exercise session (such as swimming, walking, biking) other than what you do around the house or as part of your work?

0 1 2 3 4 5 6 7

**Q.3 Blood Sugar Testing**

On how many of the last SEVEN DAYS did you test your blood sugar?

1. 1 2 3 4 5 6 7

On how many of the last SEVEN DAYS did you test your blood sugar the number of times recommended by your health care provider?

0 1 2 3 4 5 6 7

**Q.4 Foot Care**

On how many of the last SEVEN DAYS did you check your feet?

0 1 2 3 4 5 6 7

On how many of the last SEVEN DAYS did you inspect the inside of your shoes?

0 1 2 3 4 5 6 7

**Q.5 Smoking**

Have you smoked a cigarette—even one puff—during the past SEVEN DAYS?

1. No
2. Yes. *If yes*, how many cigarettes did you smoke on an average day? Number of cigarettes:

Additional Items for the Expanded Version of the Summary of Diabetes Self-Care Activities.

**Q.6 Self-Care Recommendations Font size**

1A. Which of the following has your health care team (doctor, nurse, dietitian, or diabetes educator) advised you to do? Please check all that apply:

a. Follow a low-fat eating plan

b.Follow a complex carbohydrate diet

c. Reduce the number of calories you eat to lose weight

d.Eat lots of food high in dietary fiber

e. Eat lots (at least 5 servings per day) of fruits and vegetables

f. Eat very few sweets (for example: desserts, non-diet sodas, candy bars)

g. Other (specify):

h.I have not been given any advice about my diet by my health care team.

2A. Which of the following has your health care team (doctor, nurse, dietitian or diabetes educator) advised you to do? Please check all that apply:

a. Get low level exercise (such as walk-ing) on a daily basis.

b.Exercise continuously for a least 20 minutes at least 3 times a week.

c. Fit exercise into your daily routine (for example, take stairs instead of elevators, park a block away and walk, etc.)

d.Engage in a specific amount, type, duration and level of exercise.

e. Other (specify):

f. I have not been given any advice about exercise by my health care team.

3A. Which of the following has your health care team (doctor, nurse, dietitian, or diabetes educator) advised you to do? Please check all that apply:

a. Test your blood sugar using a drop of blood from your finger and a color chart.

b.Test your blood sugar using a machine to read the results.

c. Test your urine for sugar.

d.Other (specify):

e. I have not been given any advice either about testing my blood or urine sugar level by my health care team.

4A. Which of the following medications for your diabetes has your doctor pre-scribed? Please check all that apply. a. An insulin shot 1 or 2 times a day. b.An insulin shot 3 or more times a

day. `

c. Diabetes pills to control my blood sugar level.

d.Other (specify):

e. I have not been prescribed either insulin or pills for my diabetes.

**Q.7 Diet**

5A. On how many of the last SEVEN DAYS did you space carbohydrates evenly through the day?

0 1 2 3 4 5 6 7

**Q.8 Medications**

A. On how many of the last SEVEN DAYS, did you take your recom-mended diabetes medication?

0 1 2 3 4 5 6 7

OR

B. On how many of the last SEVEN DAYS did you take your recommended insulin injections?

0 1 2 3 4 5 6 7

C. On how many of the last SEVEN DAYS did you take your recommended num-ber of diabetes pills?

0 1 2 3 4 5 6 7

**Q.9 Foot Care**

A. On how many of the last SEVEN DAYS did you wash your feet?

0 1 2 3 4 5 6 7

B. On how many of the last SEVEN DAYS did you soak your feet?

0 1 2 3 4 5 6 7

C. On how many of the last SEVEN DAYS did you dry between your toes after washing?

0 1 2 3 4 5 6 7

**Q.11 Smoking**

A. At your last doctor’s visit, did anyone ask about your smoking status?

1. No
2. Yes

B.. If you smoke, at your last doctor’s visit, did anyone counsel you about stopping smoking or offer to refer you to a stop-smoking program?

1. No
2. Yes
3. Do not smoke.

C. When did you last smoke a cigarette? More than two years ago, or never smoked

One to two years ago Four to twelve months ago One to three months ago Within the last month Today
